# Lipid Metabolic Reprogramming and Chemoresistance in Pancreatic Ductal Adenocarcinoma: Insights From Lipidomics

**DOI:** 10.1002/cbf.70333

**Published:** 2026-10-27

**Authors:** Revathi Valliyodu, Diya Maria Siril, Harsha Sanil, Areej Nazarudeen, Aswathy Usha Vijayamoorthy, Mahesh Chandran Valiyavilakathuveedu Rajan, Sudhina Sufina, Chandana Yesudas, Abhirami Nalinakshan, Radhika Achuthan, Muthunayagam Vibin, Janeesh Plakkal Ayyappan

**Affiliations:** ^1^ Department of Biochemistry University of Kerala, Kariavattom Campus Thiruvananthapuram Kerala India; ^2^ Centre for Advanced Cancer Research, Department of Biochemistry University of Kerala, Kariavattom Campus Thiruvananthapuram Kerala India; ^3^ Department of Biotechnology University of Kerala, Kariavattom Campus Thiruvananthapuram Kerala India; ^4^ CEPCI Laboratory & Research Institute (The Cashew Export Promotion Council of India) Kollam Kerala India; ^5^ DBT‐SAHAJ National Facility for Mass Spectrometry, Rajiv Gandhi Centre for Biotechnology Thiruvananthapuram India; ^6^ Department of Biochemistry Government Arts and Science College, Kulathoor Thiruvananthapuram Kerala India; ^7^ Department of Biochemistry, St. Albert's College (Autonomous) Mahatma Gandhi University Ernakulam Kerala India

**Keywords:** chemoresistance, ferroptosis, lipid metabolism, lipidomics, pancreatic cancer, PDAC

## Abstract

Pancreatic ductal adenocarcinoma (PDAC) exhibits profound resistance to chemotherapy that cannot be fully explained by mutational drivers alone. This review examines how lipid metabolic reprogramming contributes to cytotoxic chemotherapy resistance in PDAC and evaluates whether lipidomics and flux‐resolved metabolic analyses can identify functionally relevant therapeutic vulnerabilities. Current evidence on lipid metabolism in PDAC was reviewed, focusing on fatty acid uptake, de novo lipogenesis, desaturation, cholesterol metabolism, lipid droplet dynamics, fatty acid oxidation and ferroptosis regulation. Recent advances in lipidomic technologies, including LC–MS‐based lipidomics, single‐cell lipidomics, spatial lipid profiling and stable isotope tracing, were also evaluated for their ability to characterise metabolic states associated with cytotoxic chemotherapy resistance. PDAC cells resistant to cytotoxic chemotherapy undergo coordinated lipid metabolic rewiring involving enhanced lipid uptake and synthesis, altered fatty acid saturation balance, cholesterol‐rich membrane remodelling and increased lipid storage. These adaptations stabilise membrane architecture, sustain survival signalling, buffer oxidative stress and suppress ferroptosis. Tumour–stroma metabolic coupling further reinforces resistance through lipid exchange and metabolic interactions involving cancer‐associated fibroblasts and immune cells. Emerging lipidomic and flux‐resolved approaches reveal substantial intratumoural heterogeneity and identify lipid‐dependent survival states not captured by genomic or transcriptomic profiling alone. Chemoresistance in PDAC represents a dynamic lipid‐dependent metabolic phenotype driven by membrane remodelling and flux redistribution rather than isolated pathway activation. Integration of lipidomics with spatial and isotope‐resolved analyses may improve therapeutic stratification and support development of combination strategies targeting lipid metabolic vulnerabilities in PDAC.

## Significance Statement

Chemoresistance is currently one of the greatest challenges in the treatment of pancreatic ductal adenocarcinoma (PDAC). The metabolic basis of this resistance is still not fully understood. This review consolidates current evidence showing that lipid metabolic reprogramming plays a central role in enabling tumour cells to survive cytotoxic chemotherapy. By discussing emerging lipidomic technologies with key lipid‐dependent resistance mechanisms, this work highlights how metabolic adaptations that are overlooked by conventional genomic approaches can influence treatment response. A better understanding of these processes may open new opportunities for patient stratification and the development of more effective therapeutic strategies for PDAC.

**Abbreviations**
4‐HNE: 4‐hydroxynonenal
ACC: acetyl‐CoA carboxylase
ACLY: ATP‐citrate lyase
ACOT8: acyl‐CoA thioesterase 8
ACSL: acyl‐CoA synthetase long‐chain
ACSS2: acetyl‐CoA synthetase 2
ALOX: lipoxygenases
ApoE: apolipoprotein E
ATP: adenosine triphosphate
CAFs: cancer‐associated fibroblasts
CSC: cancer stem cell
DPP4: dipeptidyl‐peptidase‐4/CD26
ECM: extracellular matrix
EMT: epithelial–mesenchymal transition
ER: endoplasmic reticulum
FABPs: fatty acid binding proteins
FAO: fatty acid oxidation
FASN: fatty acid synthase
FATPs: fatty acid transport proteins
FSP1: ferroptosis suppressor protein‐1
GPX4: glutathione peroxidase‐4
GSH: glutathione
GSTP1: glutathione S‐transferase P1
HMGCR: HMG‐CoA reductase
IDH1: isocitrate dehydrogenase 1
LC‐MS: liquid chromatography–mass spectrometry
LDLR: LDL receptor
LDs: lipid droplets
LPCAT2: lysophosphatidylcholine acyltransferase‐2
m^6^A: *N* ^6^‐methyladenosine
MCT: monocarboxylate transporter
MDA: malondialdehyde
MDSCs: myeloid‐derived suppressor cells
MRD: minimal residual disease
MUFAs: monounsaturated fatty acids
NOX1: NADPH oxidase‐1
PanIN: pancreatic intraepithelial neoplasia
PC: phosphatidylcholine
PDAC: pancreatic ductal adenocarcinoma
PDGF‐C: platelet‐derived growth factor‐C
POR: cytochrome P450 oxidoreductase
PUFAs: polyunsaturated fatty acids
ROS: reactive oxygen species
S1P: sphingosine‐1‐phosphate
SCD1: stearoyl‐CoA desaturase 1
SFAs: saturated fatty acids
SOAT1: sterol O‐acyltransferase 1
SPHK1: sphingosine kinase 1
SREBP: sterol regulatory element‐binding proteins
TAGs: triacylglycerols
TAMs: tumour‐associated macrophages
TGF‐β2: transforming growth factor‐beta 2
TME: tumour microenvironment

## 1 Introduction

Pancreatic ductal adenocarcinoma (PDAC) remains among the most lethal human malignancies and constitutes the predominant histological subtype of pancreatic cancer. In 2024, approximately 67,000 new cases were diagnosed in the United States, with incidence continuing to rise annually. PDAC is currently the third leading cause of cancer‐related mortality in the United States and is projected to become the second by 2030. Globally, although ranked twelfth in incidence, it is the sixth leading cause of cancer‐related death, reflecting a disproportionate mortality burden [1, 2, 3]. In contrast to several major malignancies in which mortality has declined, survival gains in PDAC have been modest, underscoring persistent therapeutic limitations. Overall 5‐year survival is approximately 13% [4, 5].

PDAC develops through a multistep progression, from pancreatic intraepithelial neoplasia to invasive carcinoma. Oncogenic KRAS activation is an early and near‐universal event, initiating aberrant signalling through MAPK and PI3K pathways. Subsequent inactivation of tumour suppressors including CDKN2A, TP53 and SMAD4 promotes genomic instability, dysregulated cell‐cycle control and evasion of apoptosis. Transcriptomic profiling has delineated classical and basal‐like subtypes, the latter associated with dedifferentiation, aggressive biology and marked chemoresistance [2].

However, mutational burden alone does not adequately explain the aggressive clinical course or therapeutic refractoriness of PDAC [6]. The tumour microenvironment (TME) exerts profound influence on disease progression and treatment response. PDAC is characterised by a dense desmoplastic stroma enriched in extracellular matrix (ECM) components and hyaluronan, accompanied by an immunosuppressive milieu composed of tumour‐associated macrophages (TAMs), myeloid‐derived suppressor cells (MDSCs) and cancer‐associated fibroblasts (CAFs). This architectural and cellular configuration restricts drug delivery, attenuates anti‐tumour immunity and facilitates metabolic crosstalk that reinforces tumour survival [2, 7, 8].

Beyond anatomical and stromal constraints, PDAC cells display pronounced metabolic plasticity that enables adaptation to hypoxia, nutrient deprivation and cytotoxic stress. While glycolysis and amino acid metabolism have been extensively investigated, lipid metabolism has remained a comparatively underexplored determinant of therapeutic tolerance. Lipids function not merely as bioenergetic substrates but as structural and signalling entities that encode membrane architecture, regulate kinase accessibility, buffer oxidative stress and modulate ferroptosis susceptibility [9, 10, 11].

Surgical resection remains the only potentially curative modality for PDAC, yet approximately 85% of patients present with locally advanced or metastatic disease at diagnosis. Even among resected patients, recurrence exceeds 80%, driven by occult micrometastatic disease and adaptive therapeutic resistance [4, 5]. For patients with locally advanced or metastatic disease, systemic chemotherapy is the major component of treatment [12, 13, 14].

Chemotherapy resistance represents a major obstacle to effective cancer treatment, arising when tumour cells acquire mechanisms that enable them to withstand the cytotoxic effects of anticancer drugs [15]. In PDAC, the therapeutic efficacy of chemotherapy is limited by intrinsic and acquired drug resistance [12, 13, 16]. Chemoresistance in PDAC is increasingly recognised as a non‐genetic, state‐dependent phenotype encoded at the level of lipid species composition, subcellular compartmentalisation and metabolic flux redistribution rather than total lipid abundance alone.

In this review, chemoresistance primarily refers to resistance to cytotoxic chemotherapy used in PDAC. Gemcitabine receives particular emphasis because it has historically been the principal focus of PDAC chemoresistance research, with extensive evidence describing intrinsic and acquired resistance to gemcitabine [12, 13, 16]. Although treatment options have expanded to include combination regimens such as FOLFIRINOX and gemcitabine plus nab‐paclitaxel, gemcitabine‐based chemotherapy remains an important component of treatment across different disease settings [5, 14, 17, 18]. Evidence for resistance to individual agents or regimens is specified where available, while findings from other therapeutic contexts are considered separately and are not assumed to represent equivalent resistance mechanisms. This review examines the current understanding of lipid metabolic reprogramming in PDAC and evaluates how advanced lipidomics, particularly flux‐resolved and single‐cell approaches, can provide mechanistic insight into resistance states that remain poorly captured by genomic or transcriptomic profiling.

## 2 Summary

Cytotoxic chemoresistance remains a major obstacle in PDAC. This review highlights how lipid metabolic reprogramming supports cytotoxic drug resistance through alterations in lipid uptake, synthesis, membrane remodelling, ferroptosis regulation and TME interactions. It further discusses how emerging lipidomic and flux‐resolved approaches reveal metabolically distinct resistant states that are not captured by genomic analyses. Understanding these lipid‐dependent adaptations may facilitate the development of mechanism‐based therapeutic strategies and improve patient stratification in PDAC.

## 3 Metabolic Plasticity in Chemoresistance

PDAC develops within a hypovascular, desmoplastic microenvironment characterised by nutrient deprivation, hypoxia and chronic metabolic stress. Chemotherapy further intensifies energetic and redox stress, while resistance to commonly used regimens such as gemcitabine‐based chemotherapy and FOLFIRINOX arises predominantly through coordinated metabolic adaptation rather than isolated genetic alterations [6, 7, 9, 19, 20].

Under therapeutic pressure, PDAC cells redistribute carbon flux across glucose, glutamine, acetate and lipid pathways to support biosynthetic and cytoprotective functions instead of complete oxidative metabolism, as illustrated in Figure 1. Genome‐scale metabolic modelling demonstrates that this adaptation reflects network‐level metabolic rewiring orchestrated by oncogenic KRAS and PI3K–AKT–mTOR signalling [19, 21]. These changes are context‐dependent and not fully captured by transcriptomic analyses alone, as transcriptional output does not directly reflect substrate routing, lipid remodelling or compartment‐specific redistribution.

**Figure 1:**
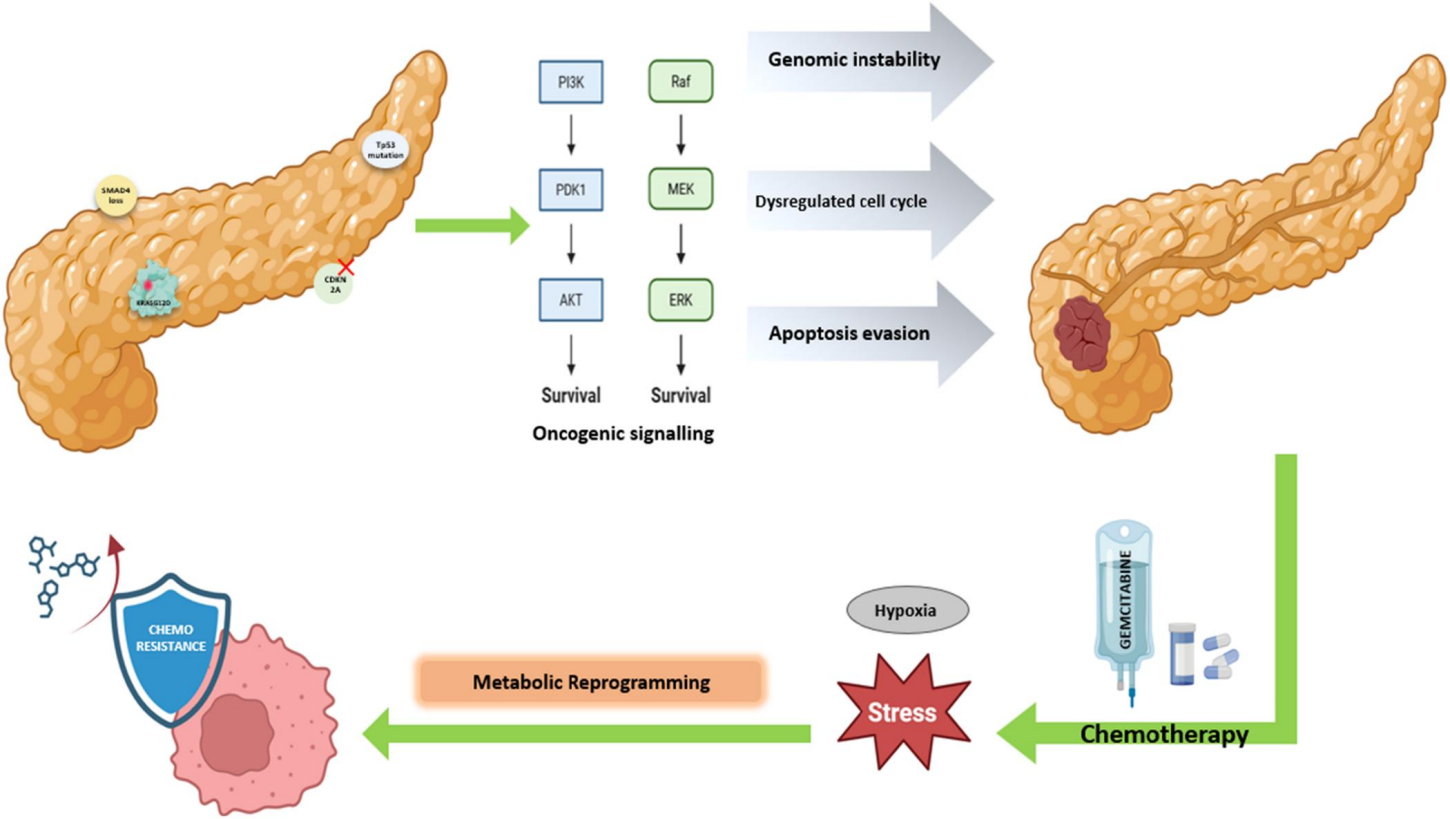
Signalling‐driven metabolic plasticity in PDAC cytotoxic chemoresistance. Oncogenic signalling and microenvironmental stress promote metabolic flux redistribution across multiple nutrient pathways, supporting tumour survival and chemoresistance [6, 19, 21].

Within this adaptive framework, lipid metabolism occupies a central role. Lipids function not only as energy substrates but also as structural and signalling components required for membrane biogenesis, lipid raft formation, organelle integrity and redox buffering [6, 9, 10, 21, 22, 23]. Chemoresistant PDAC cells frequently enhance lipid remodelling, storage and alternative substrate utilisation while retaining functional mitochondrial metabolism [4, 5, 11, 24]. Rather than maximising ATP production, lipid‐derived carbon is often redirected towards membrane maintenance, neutral lipid storage and oxidative stress tolerance.

Therapy‐induced lipid remodelling is selective and pathway‐specific, involving alterations in glycerophospholipids, sphingolipids and fatty acyl species associated with treatment response [5, 25, 26, 27]. Collectively, these adaptations establish a durable metabolic phenotype characterised by altered lipid composition, saturation balance and flux redistribution that supports membrane stability, survival signalling and resistance to cytotoxic stress.

## 4 Major Lipid Reprogramming Axes in Cytotoxic Chemotherapy Resistance in PDAC

Lipid metabolic reprogramming in PDAC represents a coordinated restructuring of fatty acid uptake, de novo lipogenesis, desaturation, cholesterol metabolism, lipid storage and oxidation rather than a uniform increase in lipid synthesis. These axes, as illustrated in Figure 2, collectively regulate membrane composition, lipid‐mediated signalling, redox homoeostasis and ferroptotic susceptibility. Under therapeutic pressure, their integration enables metabolic flexibility, supports cytoprotective adaptations such as lipid droplet formation and antioxidant defence, and stabilises chemoresistant phenotypes.

**Figure 2:**
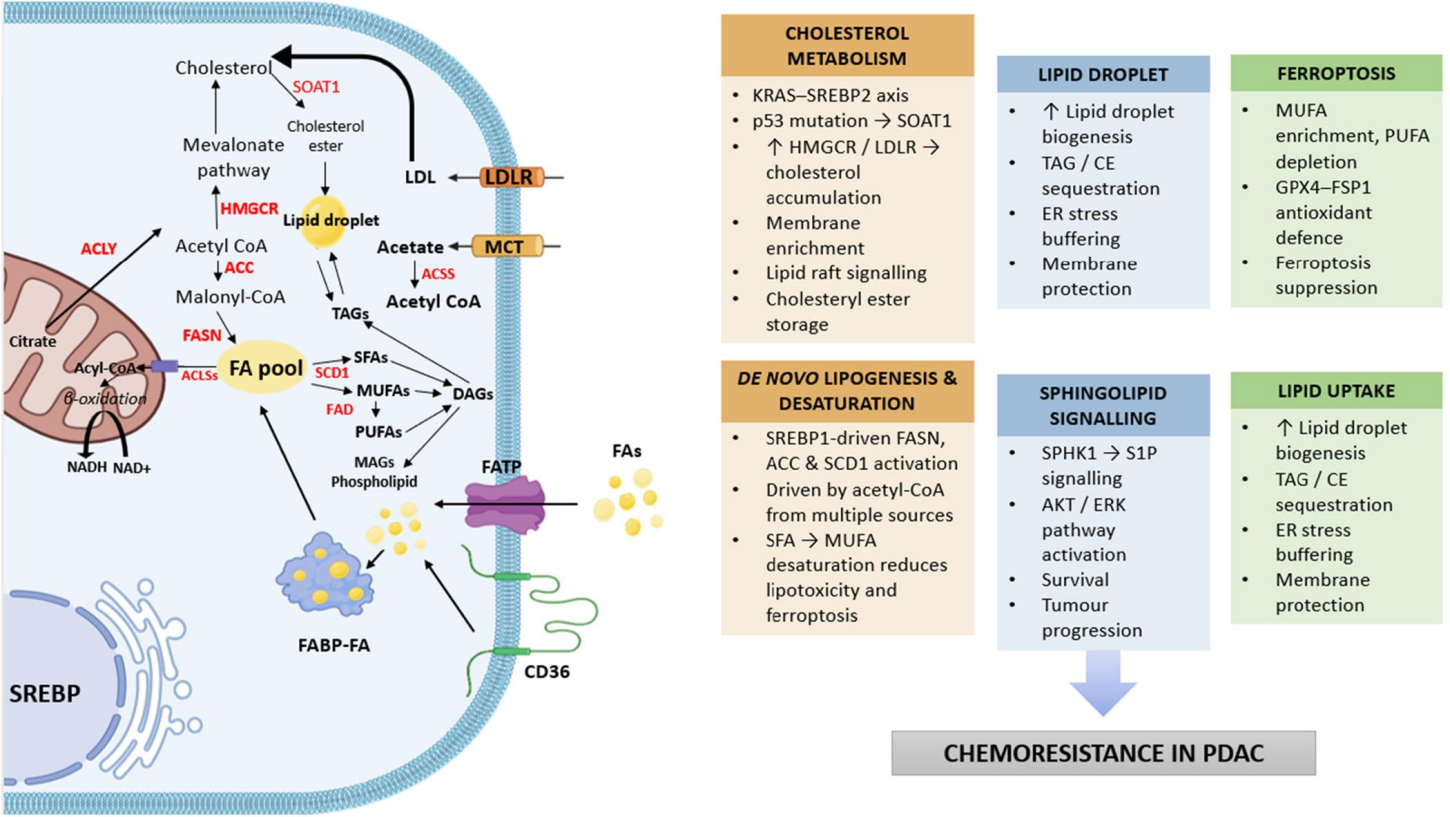
Integrated lipid metabolic reprogramming in chemoresistant PDAC. Coordinated regulation of lipid uptake, synthesis, desaturation, cholesterol metabolism and lipid storage remodels membrane composition, maintains redox homoeostasis and suppresses ferroptosis, thereby promoting tumour survival and chemoresistance [9, 21, 23, 28, 29].

### 4.1 Lipid Uptake and Exogenous Substrate Integration

Extracellular fatty acid availability directly influences lipid metabolic behaviour in PDAC. Under hypoxic conditions, reliance on exogenous lipid uptake increases, enabling preferential incorporation of monounsaturated fatty acids (MUFAs) into membrane phospholipids to preserve membrane fluidity while limiting lipid peroxidation [9, 23, 30].

PDAC cells exhibit high rates of fatty acid uptake mediated by CD36, fatty acid transport proteins (FATPs) and fatty acid binding proteins (FABPs). CD36‐mediated lipid uptake supports metabolic adaptation under nutrient limitation and therapeutic stress while also contributing to metastatic progression [8, 9]. CPT1C additionally regulates mitochondrial fatty acid handling, and its inhibition promotes compensatory CD36 upregulation and intracellular lipid accumulation, highlighting the dynamic balance between fatty acid uptake and oxidation [31].

Fatty acid species composition also influences cellular behaviour. Alterations in palmitate, stearate and omega‐3/omega‐6 phospholipid species modify membrane architecture and downstream signalling pathways. Stearate suppresses proliferation and induces senescence, whereas oleate restores proliferative capacity in multiple cancer models, including PANC‐1 cells [28, 31]. These findings indicate that lipid composition, rather than total lipid abundance, determines functional metabolic outcomes.

### 4.2 De Novo Lipogenesis and Fatty Acid Desaturation

Despite substantial lipid uptake, oncogenic KRAS activation drives robust de novo lipogenesis in PDAC [21, 23]. Under hypoxia, non‐canonical pathways including IDH1‐mediated reductive carboxylation sustain fatty acid synthesis. Multiple carbon sources converge on acetyl‐CoA, including glucose‐derived pyruvate, glutamine, acetate, citrate and lactate‐derived carbons, thereby maintaining lipid synthesis under nutrient‐limited conditions [5].

SREBP1 and SREBP2 coordinate transcriptional regulation of fatty acid and cholesterol synthesis. SREBP1 induces expression of fatty acid synthase (FASN), acetyl‐CoA carboxylase (ACC) and stearoyl‐CoA desaturase 1 (SCD1), while PDGF‐C further amplifies lipogenic signalling [8, 9, 21, 23, 28, 32, 33]. FASN supports membrane biogenesis, ER stress adaptation, EMT and stemness phenotypes, whereas SCD1‐mediated conversion of saturated fatty acids (SFAs) to MUFAs preserves membrane fluidity and limits lipotoxicity [5, 9, 21, 23, 26, 28, 32, 34, 35].

Desaturation balance is central to ferroptosis resistance. MUFA enrichment reduces incorporation of highly peroxidation‐prone polyunsaturated fatty acids (PUFAs) into membrane phospholipids, while altered SFA/MUFA and PUFA/MUFA ratios influence membrane organisation and signalling. Membrane lipid composition promotes AKT recruitment, phosphoinositide clustering and lipid raft stabilisation, thereby sustaining pro‐survival signalling independent of additional genetic alterations [9, 21, 23, 26, 35, 36].

### 4.3 Cholesterol Metabolism

Cholesterol metabolism functions as a membrane‐centred resistance mechanism in PDAC. KRAS‐driven SREBP2 activation enhances cholesterol biosynthesis and LDL receptor (LDLR)‐mediated uptake through regulation of HMG‐CoA reductase (HMGCR) and LDLR expression [9, 21, 23, 28, 36]. Sterol O‐acyltransferase 1 (SOAT1), which converts free cholesterol into cholesterol esters, is upregulated in TP53‐mutant PDAC and supports lipid storage and tumour growth [37, 38].

Accumulation of cholesteryl esters and increased cholesterol incorporation into plasma and organelle membranes enhance membrane order, reduce drug permeability and stabilise lipid raft microdomains that sustain ERK, AKT, STAT3 and Hedgehog signalling [21, 23, 27, 34, 39]. Cholesterol metabolism additionally contributes to acinar‐to‐ductal metaplasia and stromal immune suppression mediated by ApoE signalling [37].

### 4.4 Lipid Droplet Dynamics

Lipid droplets (LDs) are dynamic organelles central to lipid buffering and metabolic flexibility. Chemotherapy, hypoxia and oxidative stress induce LD biogenesis in PDAC, and LD accumulation correlates with tumour aggressiveness and poor prognosis [9, 10, 19, 21, 27, 34, 35, 40].

LDs sequester triacylglycerols and sterol esters derived from endogenous or exogenous fatty acids. By storing PUFAs within neutral lipid pools, LDs suppress lipid ROS accumulation and ferroptotic susceptibility while limiting ER stress and mitochondrial dysfunction [4, 9, 35, 40]. Stored fatty acids can subsequently be mobilised for β‐oxidation or membrane remodelling during acute stress.

LPCAT2, a phosphatidylcholine remodelling enzyme, promotes lipid droplet formation and contributes to proliferation, invasion and gemcitabine resistance in pancreatic cancer cells [41]. Expansion of lipid storage capacity therefore reflects adaptive flux redistribution rather than simple lipid accumulation.

### 4.5 Fatty Acid Oxidation

Fatty acid oxidation (FAO) in PDAC is context‐dependent and contributes to energy homoeostasis under metabolic stress. ACSL family members such as ACSL3 and ACSL4 facilitate fatty acid activation and mitochondrial utilisation [9, 21]. Although glycolytic reprogramming is prominent, chemoresistant PDAC retains active mitochondrial metabolism and frequently redirects lipid‐derived carbon towards biosynthesis and neutral lipid storage rather than complete oxidative degradation [4, 5, 11, 24]. Modulation of peroxisomal β‐oxidation and carnitine‐dependent mitochondrial transport therefore reflects prioritisation of membrane maintenance and redox balance over maximal ATP production.

### 4.6 Ferroptosis Defence

Complete suppression of lipid peroxidation is uncommon in PDAC. Instead, tumour cells maintain sublethal lipid ROS levels that function as adaptive signalling cues while avoiding ferroptotic cell death [27, 35]. Resistance‐associated lipid states are typically characterised by MUFA enrichment, PUFA depletion and enhanced antioxidant capacity, particularly through activation of the glutathione peroxidase 4 (GPX4) pathway. GPX4 reduces phospholipid hydroperoxides and functions as a central suppressor of ferroptosis, while oncogenic signalling pathways including p53‐ and NRF2‐mediated responses further reinforce oxidative stress tolerance [25, 27, 35, 42, 43].

Additional antioxidant systems operate in parallel with GPX4. Ferroptosis suppressor protein‐1 (FSP1) regenerates ubiquinol and suppresses membrane lipid peroxidation independently of GPX4, promoting resistance to gemcitabine and maintaining lipid homoeostasis [29, 44]. Elevated ACOT8 expression similarly shifts lipid composition towards MUFA dominance and suppresses ferroptosis, whereas cholesterol intermediates such as 7‐dehydrocholesterol additionally influence membrane oxidative dynamics [9, 35, 45].

Membrane phospholipid composition is a major determinant of ferroptosis susceptibility. PUFA‐containing ether phospholipids are highly prone to lipid peroxidation, and disruption of the peroxisomal ether‐lipid biosynthetic pathway reduces ferroptosis sensitivity by limiting PUFA incorporation into membrane phospholipids [46]. The TME further modulates ferroptosis through CAF‐derived exosomal miR‐3173‐5p, which suppresses ACSL4‐mediated PUFA incorporation and promotes gemcitabine resistance [47].

Together, these findings indicate that ferroptosis susceptibility in PDAC is governed by coordinated regulation of lipid desaturation, membrane phospholipid composition, lipid storage pathways and antioxidant defence systems.

### 4.7 Sphingolipid Signalling

Sphingolipid remodelling contributes to lipid‐mediated resistance and stem‐like phenotypes in PDAC. Upregulation of sphingosine kinase 1 (SPHK1) increases sphingosine‐1‐phosphate (S1P) production, activating AKT and ERK signalling pathways that enhance tumoursphere formation and suppress apoptosis [48]. Enhanced glycosphingolipid biosynthesis additionally supports KRAS membrane localisation and downstream signalling [9].

Lipid metabolic rewiring also suppresses pro‐apoptotic signalling through inhibition of factors such as Bad, Bim and FoxO while stabilising receptor clustering and membrane‐associated survival signalling [5, 34]. Autophagy further functions as a metabolic buffering system that supplies fatty acids and biosynthetic substrates under chemotherapy‐induced stress, thereby supporting tumour survival and metabolic flexibility [5].

Redox regulation is closely integrated with lipid metabolism through signalling networks involving NRF2, HIF‐1α and SREBP [49]. NADPH‐dependent lipid synthesis, glutathione cycling and GSTP1‐mediated redox regulation collectively reinforce oxidative stress resilience and maintain lipid homoeostasis under chemotherapeutic stress [11, 50, 51].

## 5 Lipid Metabolism Within the Tumour Ecosystem

Lipid metabolic rewiring in PDAC does not occur in isolation within malignant epithelial cells but is shaped by stromal interactions, immune modulation and spatial metabolic gradients within a desmoplastic and hypovascular TME. Resistance therefore reflects coordinated metabolic coupling between tumour cells and their surrounding ecosystem rather than purely tumour‐intrinsic pathway activation [52].

Tumour‐derived metabolites, including lactate and lipid species, contribute to extracellular acidification that promotes EMT, CAF activation and immunosuppressive niche formation. Concurrently, impaired perfusion and hypoxia stabilise HIF1α signalling, driving angiogenesis and reprogramming lipid uptake, storage and remodelling pathways associated with chemoresistance, as illustrated in Figure 3 [7, 19, 53]. Lipid metabolites released from tumour and stromal compartments further modulate immune cell metabolism through pathways involving CD36, CPT1A, FASN and SCD1, reinforcing immune suppression and therapeutic resistance [21].

**Figure 3:**
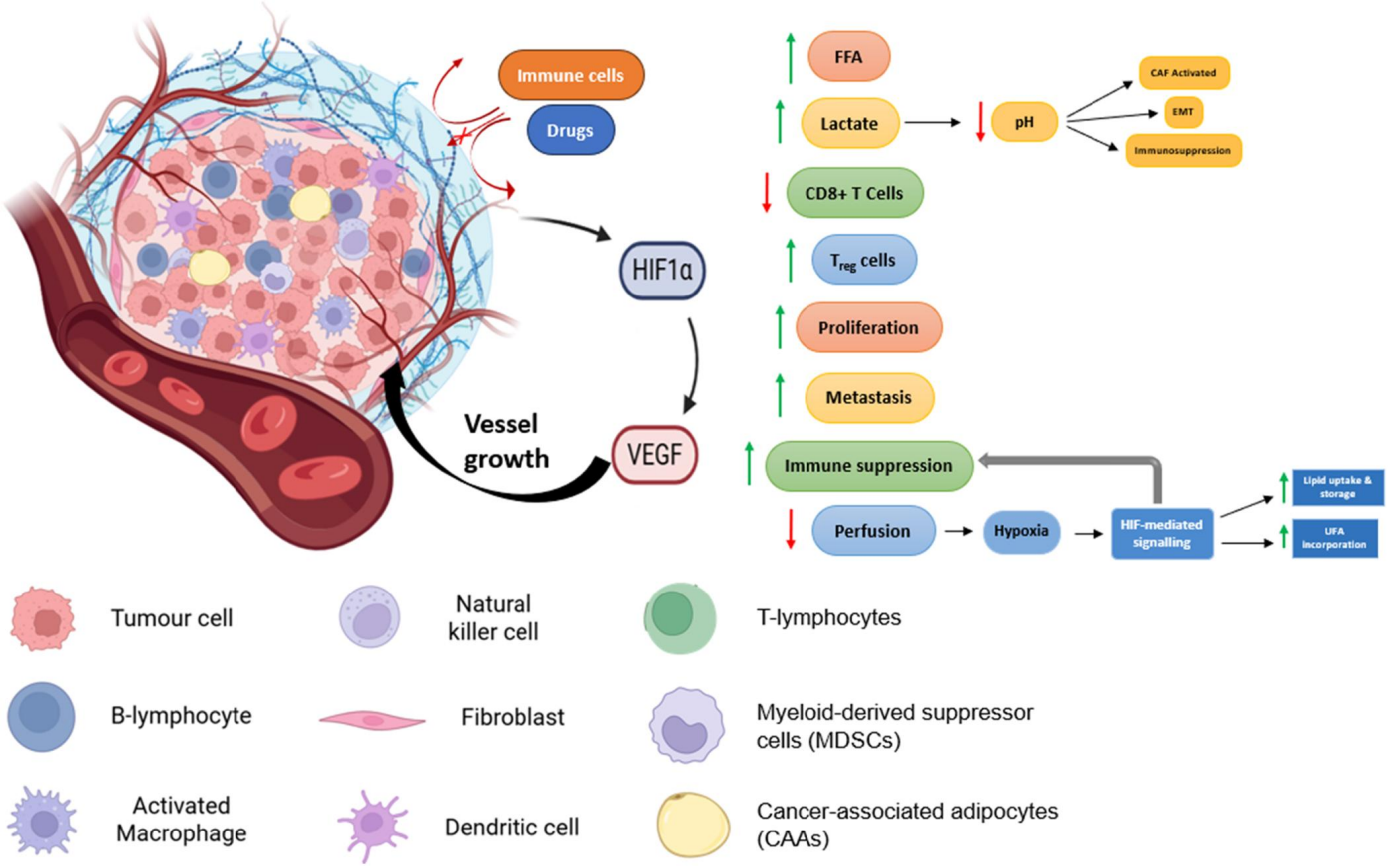
Tumour microenvironmental regulation of chemoresistance in PDAC. Hypoxia, extracellular acidification and stromal interactions promote lipid metabolic reprogramming, immune suppression and survival signalling within the tumour microenvironment, thereby supporting chemoresistance [7, 19, 20, 51, 53].

### 5.1 Metabolic Zonation

The PDAC lipidome is environmentally responsive rather than genetically fixed. Oxygen gradients, nutrient deprivation and extracellular fatty acid availability dynamically regulate de novo lipogenesis, lipid remodelling and exogenous lipid uptake. Hypoxic niches preferentially promote lipid scavenging and incorporation of UFAs, preserving membrane fluidity and adaptive stress tolerance [9, 30].

Spatial multi‐omics and single‐cell lipidomic analyses reveal pronounced intratumoural heterogeneity, including glycolytic, fatty acid‐dependent and metabolically flexible subpopulations shaped by local microenvironmental constraints, as illustrated in Figure 4 [5, 6, 9, 54, 55].

**Figure 4:**
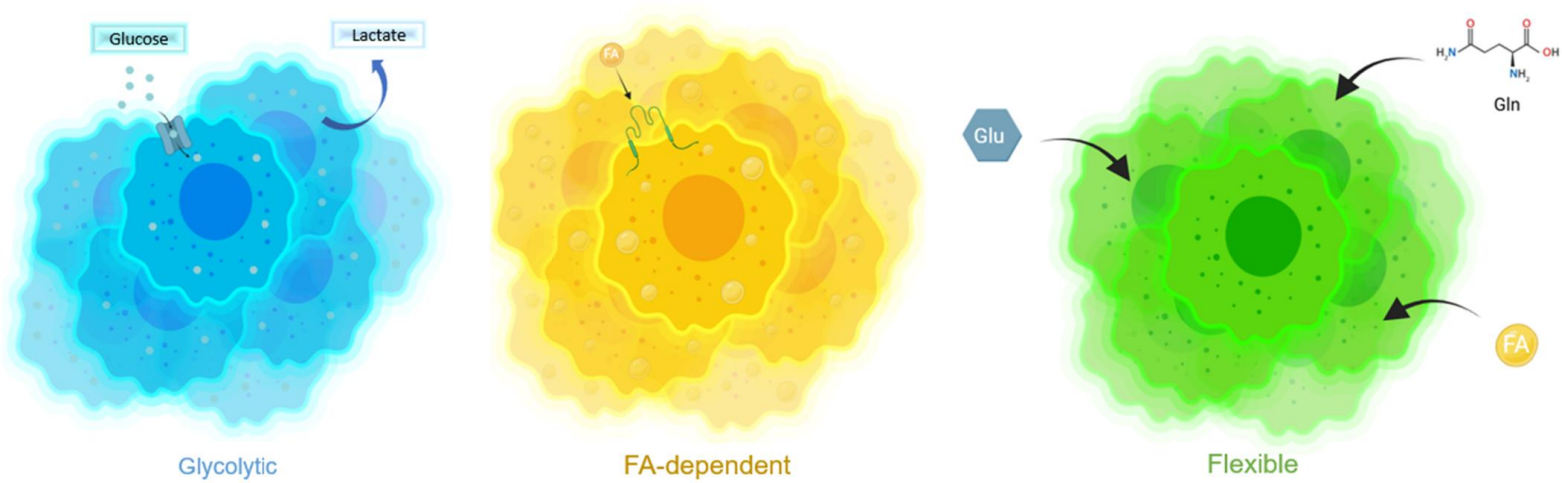
Intratumoural metabolic heterogeneity in PDAC. Spatially distinct tumour cell populations exhibit diverse metabolic phenotypes that enable adaptive metabolic switching and formation of chemoresistant niches [5, 6, 9, 54, 55].

Chemotherapeutic pressure further selects for lipid‐enriched, membrane‐stabilised and stress‐tolerant subpopulations characterised by altered SFA/MUFA/PUFA ratios and reduced oxidative and ER stress susceptibility, while lipid‐depleted populations undergo metabolic collapse, as illustrated in Figure 5 [5, 48].

**Figure 5:**
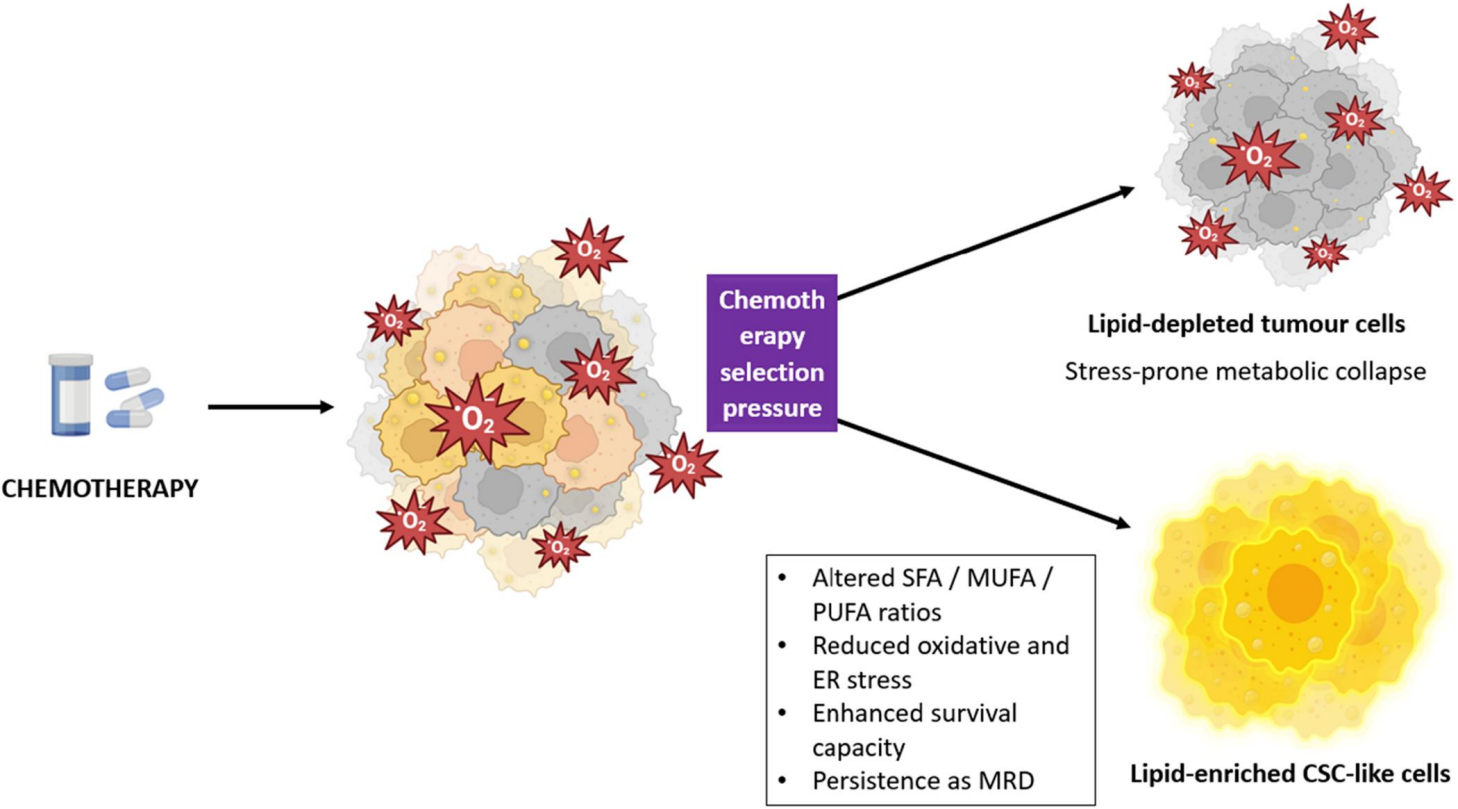
Chemotherapy‐driven selection of lipid‐adapted PDAC subpopulations. Chemotherapeutic stress selects for lipid‐enriched, stress‐tolerant tumour cells with altered fatty acid composition and enhanced survival capacity, contributing to chemoresistance [5, 48, 55].

These findings indicate that resistance reflects metabolic selection under environmental pressure, leading to enrichment of lipid‐adapted tumour cell states that persist as minimal residual disease following therapy [5, 53, 55].

### 5.2 Cancer Stem Cell Maintenance and Lipid‐Driven Immunosuppression

Lipid metabolic rewiring is closely linked to CSC maintenance in PDAC. CSC populations exhibit enhanced de novo fatty acid synthesis, increased desaturation, expanded neutral lipid storage and reinforced ferroptosis resistance [9, 42, 53, 56]. These adaptations support self‐renewal, survival under metabolic stress and long‐term persistence following chemotherapy. MUFA enrichment and lipid droplet accumulation buffer oxidative stress and stabilise membrane integrity, thereby limiting apoptosis and ferroptosis [27, 56].

TME‐driven pathways sustaining CSC populations, including TGF‐β, WNT/β‐catenin and Notch signalling, intersect with SREBP‐mediated lipogenic programmes and sphingolipid signalling networks [42, 48, 53]. SPHK1‐driven S1P production further promotes AKT/ERK activation and stem‐like phenotypes [48].

Lipid accumulation within immune cells additionally contributes to an immunosuppressive microenvironment. Lipid‐laden macrophages adopt pro‐tumourigenic phenotypes and impair T‐cell function, while FABP7‐dependent lipid accumulation enhances ECM remodelling and metastatic niche formation [8, 10, 20, 21, 28, 55]. Lipid metabolites and extracellular vesicles mediate bidirectional communication between tumour, stromal and immune populations, reinforcing immune evasion and drug tolerance [9, 10].

### 5.3 Cancer‐Associated Fibroblasts

CAFs function as lipid reservoirs within the PDAC microenvironment. CAFs release fatty acids, lysophospholipids, lipid droplets and extracellular vesicle‐associated lipids that enhance tumour lipid uptake, esterification and lipid droplet biogenesis while upregulating transporters such as FATPs and CD36 [20, 51]. CAF‐mediated lipid transfer also supports β‐oxidation, de novo lipogenesis, EMT and membrane remodelling.

In addition, CAFs influence tumour autophagy and lipophagy, sustaining survival when chemotherapy disrupts metabolic homoeostasis. Lipid mediator signalling contributes to ECM stiffening and maintenance of an immunosuppressive niche, including prostaglandin E2 production [51]. Disruption of CAF‐to‐tumour lipid transfer reduces tumour viability and sensitises cells to metabolic and cytotoxic stress, highlighting the importance of lipid‐mediated intercellular networks in maintaining chemoresistance [20].

## 6 Epigenetic and Regulatory Control of Lipid Metabolism in PDAC

The lipid metabolic configuration observed in chemoresistant PDAC is not a passive by‐product of oncogenic signalling but an actively maintained transcriptional and post‐transcriptional state. Multiple regulatory layers converge to stabilise lipogenic flux, desaturation balance, lipid storage capacity and ferroptosis resistance. These nodes function as adjustable control points that determine lipid carbon fate and sustain survival under therapeutic pressure.

### 6.1 Epigenetic Regulation via the TET3–GATA6 Axis

An additional regulatory layer involves epigenetic control of lipogenic transcriptional programmes.

GATA6 is a zinc‐finger transcription factor essential for pancreatic development and lineage specification. In PDAC, GATA6 amplification characterises the classical subtype, where it functions as a lineage‐specific oncogene while maintaining epithelial identity and repressing basal‐like transcriptional programmes. Through inhibition of EMT, GATA6 constrains phenotypic plasticity [57, 58].

TET3 has emerged as a key regulator of lipogenic metabolism in PDAC and is enriched in lipogenic tumour subtypes, correlating with poor prognosis. Genetic ablation of TET3 disrupts lipid homoeostasis, leading to altered lipid droplet accumulation, shifts in SFA/MUFA ratios and broad perturbations in lipid metabolic profiles [58].

TET3 epigenetically represses GATA6 through recruitment of histone deacetylases to the GATA6 promoter. This repression sustains high expression of genes involved in fatty acid synthesis, desaturation and lipid storage, including SCD1 and ACSL3. Lipidomic profiling of TET3‐deficient PDAC cells reveals altered fatty acid species distribution and increased oxidative susceptibility due to derepression of GATA6. Disruption of the TET3‐GATA6 axis sensitises cells to ferroptosis, directly linking epigenetic regulation to lipid metabolic rewiring [58].

Therapeutically, combination strategies targeting this axis are mechanistically rational. Histone deacetylase inhibitors combined with ferroptosis inducers such as erastin, alongside gemcitabine, markedly enhance cytotoxicity in lipogenic PDAC models. This approach destabilises lipogenic identity while simultaneously increasing lipid peroxidation stress [10, 58].

### 6.2 Post‐Transcriptional Regulation by m^6^A‐Modified TGF‐β2

Post‐transcriptional regulation further contributes to lipid‐driven chemoresistance. *N* ^6^‐methyladenosine (m^6^A) modification of transforming growth factor‐beta 2 (TGF‐β2) mRNA increases transcript stability and enhances TGF‐β2 expression in PDAC cells. Elevated TGF‐β2 functions as an upstream driver of lipid metabolic reprogramming, linking epitranscriptomic regulation to metabolic adaptation [59].

TGF‐β2 activation induces a lipogenic phenotype characterised by increased lipid accumulation and reprogrammed fatty acid metabolism that favours survival under drug stress. Downstream signalling cascades converge on lipid metabolic regulators, integrating cytokine signalling with metabolic control. TGF‐β2‐mediated lipid rewiring reduces gemcitabine‐induced cytotoxicity and high TGF‐β2 expression correlates with gemcitabine resistance and poor therapeutic response [59].

Pharmacological or genetic inhibition of lipid metabolic pathways partially reverses TGF‐β2‐driven gemcitabine resistance, establishing a causal link between lipid reprogramming and therapeutic failure. Targeting m^6^A regulatory machinery, TGF‐β2 signalling or downstream lipid metabolic effectors sensitises PDAC cells to chemotherapy. TGF‐β2‐associated lipid signatures therefore hold potential as predictive biomarkers for treatment response [59].

## 7 Lipidomics and Flux‐Resolved Analysis for Chemoresistance Mapping

Genomic and transcriptomic profiling do not fully capture the stable metabolic states associated with chemoresistance in PDAC. Resistance frequently manifests through altered lipid species composition, membrane organisation, subcellular lipid compartmentalisation and redistribution of carbon flux rather than transcriptional changes alone [3, 6, 9, 60]. Lipidomics, predominantly based on LC–MS platforms, enables characterisation of lipid alterations across classes and molecular species using targeted and untargeted approaches [61, 62, 63, 64].

However, conventional bulk lipidomics primarily quantifies steady‐state lipid abundance and provides limited mechanistic insight into pathway activity. Identical lipid pools may arise from altered synthesis, degradation, turnover or acyl‐chain remodelling [5, 6, 65]. Resistance‐associated lipid rewiring in PDAC is frequently qualitative rather than quantitative, involving changes in fatty acid saturation, membrane microdomain organisation and lipid compartmentalisation without major changes in total lipid abundance [27, 35, 48]. Bulk tumour measurements also obscure metabolically distinct tumour, stromal and immune cell populations within heterogeneous PDAC tissues [9, 55, 66].

Single‐cell and spatial lipidomics overcome these limitations by resolving intratumoural metabolic heterogeneity and identifying lipid‐defined subpopulations associated with cytotoxic chemotherapy resistance, as illustrated in Figure 6. Chemotherapy‐treated PDAC contains metabolically distinct lipid‐enriched and lipid‐depleted cell states associated with differences in phospholipid remodelling, SFA/MUFA/PUFA balance and oxidative stress tolerance [5, 9, 48, 67]. Functionally distinct high‐synthesis, quiescent and lipid‐scavenging phenotypes may coexist within the same TME [67, 68].

**Figure 6:**
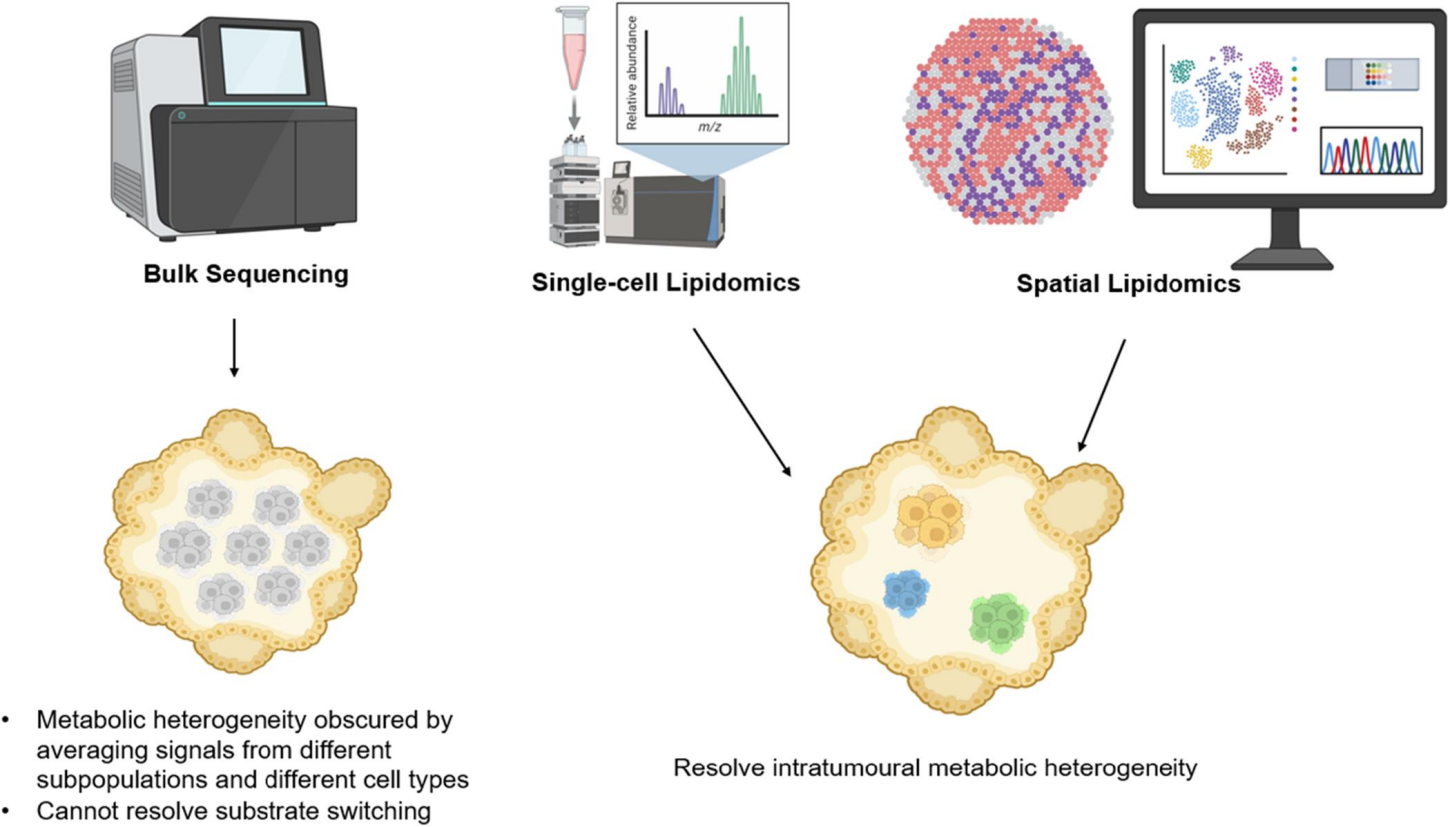
Resolution of intratumoural metabolic heterogeneity by single‐cell and spatial lipidomics. Single‐cell and spatial lipidomic approaches resolve distinct tumour metabolic states and lipid dependencies that are obscured by bulk analyses [9, 55, 66, 67].

Stable isotope tracing further extends lipidomic analysis beyond abundance measurements by quantifying lipid synthesis, turnover, desaturation and substrate utilisation. Incorporation of labelled substrates such as ^13^C‐glucose, ^13^C‐glutamine, ^13^C‐acetate and labelled fatty acids enables direct assessment of de novo lipogenesis, reductive carboxylation, cholesterol synthesis and exogenous lipid uptake [52, 65, 66, 69, 70, 71, 72]. Isotopologue‐resolved lipidomics distinguishes newly synthesised lipids from pre‐existing pools and reveals adaptive flux redistribution that may occur without detectable changes in steady‐state abundance [52, 65, 66, 71, 72, 73, 74].

Multi‐substrate and co‐culture tracing approaches further demonstrate coexistence of distinct metabolic programmes within PDAC and reveal stromal‐tumour metabolite exchange that shapes resistance phenotypes [9, 20, 71, 73, 75]. Together, species‐resolved lipidomics, spatial analysis and isotope‐resolved flux tracing provide a functional framework for identifying lipid metabolic states associated with cytotoxic chemotherapy resistance beyond conventional genomic classification.

### 7.1 An Integrated Flux Framework for Decoding Resistance

Resolving lipid‐driven chemoresistance in PDAC requires integration of complementary analytical and functional approaches, including:

- Species‐resolved lipidomics.
- Single‐cell and spatial resolution.
- Stable isotope tracing.
- Flux modelling.
- Functional perturbation studies.

Together, these modalities provide complementary layers of information spanning lipid composition, spatial heterogeneity, substrate utilisation and pathway activity. Only this integrated framework captures how lipid metabolic rewiring sustains survival, propagates resistance and escapes detection by steady‐state metabolomics. The contribution of each approach to resolving metabolic heterogeneity and flux is illustrated in Figure 7.

**Figure 7:**
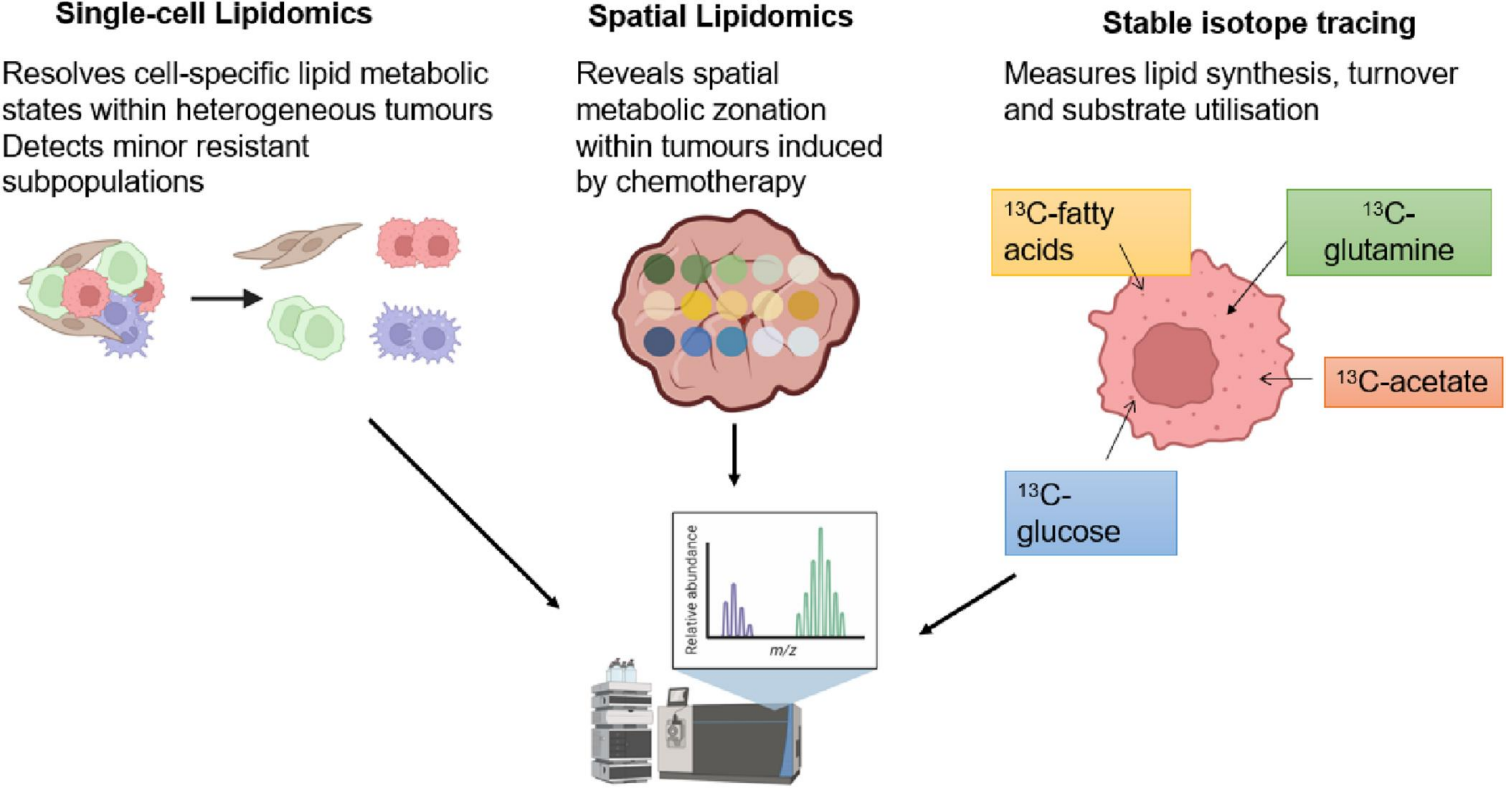
Integrated framework for resolving lipid metabolic heterogeneity and flux in PDAC. Single‐cell, spatial and isotope‐resolved lipidomic approaches collectively define lipid metabolic states, substrate utilisation and flux rewiring associated with chemoresistance [52, 65, 66, 71].

In this context, lipidomics extends beyond a descriptive inventory of metabolites to function as a mechanistic readout of cellular survival capacity.

## 8 Therapeutic Targeting of Lipid‐Driven Cytotoxic Chemotherapy Resistance

Lipid metabolism represents a potential therapeutic vulnerability in PDAC, particularly when combined with cytotoxic chemotherapy. Current evidence is predominantly preclinical and is strongest for sensitisation to gemcitabine‐based treatment.

Targeting lipid metabolic pathways can impair tumour growth, sensitise malignant cells to cytotoxic chemotherapy and disrupt metabolic flexibility associated with adaptive resistance [23]. However, clinical translation of lipid‐directed therapies has been limited, reflecting the networked and compensatory nature of lipid metabolism within tumour and stromal compartments [9, 76].

A mechanistically coherent therapeutic strategy requires recognising that lipid metabolism in PDAC is not a single pathway but a coordinated system governing membrane architecture, redox balance, ferroptosis sensitivity and signalling robustness.

### 8.1 Targeting Lipid Uptake and Tumour–Stroma Coupling

CD36‐mediated fatty acid uptake and CAF‐driven lipid transfer reinforce tumour metabolic flexibility [8, 20, 51]. Inhibition of lipid uptake reduces lipid droplet accumulation, limits β‐oxidation and disrupts stromal–tumour metabolic coupling.

However, tumour cells can compensate by increasing de novo synthesis or altering substrate utilisation [20]. Effective targeting may therefore require simultaneous disruption of uptake and synthesis pathways, particularly in lipid‐rich microenvironments.

Given the ecosystem‐level organisation of lipid metabolism in PDAC, tumour‐intrinsic targeting without accounting for stromal and immune lipid support is unlikely to produce sustained benefit.

### 8.2 Targeting De Novo Lipogenesis and Desaturation

De novo lipogenesis is sustained in PDAC through activation of ACC, FASN and SCD1 [9, 23, 28, 32]. These enzymes also maintain saturation balance and reinforce pro‐survival signalling.

Pharmacological inhibition of FASN reduces stem‐like features, disrupts ER stress adaptation and partially restores sensitivity to gemcitabine and platinum‐based agents [5, 28, 32, 34]. Inhibition of ACC or ACLY similarly reduces cytosolic acetyl‐CoA availability, limiting fatty acid and cholesterol synthesis [9, 19, 23]. Inhibition or knockdown of PDGF‐C or SREBP1 reduces lipid synthesis and suppresses PDAC cell proliferation and invasiveness [33].

Overexpression of SCD1 protects PDAC cells from apoptosis and ferroptosis under hypoxia and chemotherapeutic stress. Pharmacological inhibition of SCD1 shifts SFA/MUFA balance, increases oxidative vulnerability and enhances drug sensitivity [9, 21, 23, 26, 35].

Despite strong preclinical rationale, single‐enzyme inhibition often triggers compensatory lipid uptake or redistribution of flux towards alternative pathways. Thus, lipogenesis targeting alone rather than combination therapies rarely yields durable responses.

### 8.3 Disrupting Mitochondrial Lipid Metabolism

Cancer cells, including PDAC, often display elevated mitochondrial membrane potential. When coupled with increased fatty acid uptake, this creates an opportunity to exploit lipid moieties as targeted delivery vectors to selectively accumulate therapeutic agents within tumour mitochondria. Disruption of mitochondrial lipid handling compromises oxidative phosphorylation and perturbs mitochondrial protein homoeostasis, leading to collapse of bioenergetic integrity and increased vulnerability to stress [24].

### 8.4 Targeting Cholesterol Flux and Membrane Architecture

Cholesterol metabolism contributes to membrane rigidity, drug permeability modulation and lipid raft‐mediated signalling stabilisation [21, 23, 27, 34, 39]. Targeting cholesterol flux alters resistance phenotypes. Silencing LDLR or inhibiting cholesterol biosynthesis reduces ERK signalling and enhances gemcitabine sensitivity [9, 19, 28]. Blocking LDL aggregation impairs tumour proliferation and reduces cholesteryl ester accumulation without directly inhibiting canonical lipid synthesis enzymes [39]. Limiting cholesteryl ester loading via SOAT1 inhibition attenuates membrane‐based resistance mechanisms, particularly in p53‐mutant contexts. Limiting cholesteryl ester loading by the pharmacological inhibition of SOAT1, using drugs such as avasimibe, suppresses proliferation of PDAC cells, with stronger effects observed in TP53‐mutant cells. SOAT1 inhibition induces cell‐cycle suppression specifically in TP53‐mutant pancreatic cancer cells, indicating a mutation‐specific metabolic vulnerability [9, 38].

Statins and HMG‐CoA‐targeted approaches have been explored clinically, but systemic toxicity, incomplete tumour specificity and metabolic compensation have limited durable benefit [9, 76]. Cholesterol flux can be restored through uptake, esterification or redistribution across membrane compartments, highlighting the need for combinatorial approaches.

### 8.5 Disrupting Lipid Droplet Dynamics

Lipid droplets function as metabolic buffers that protect against oxidative stress, ferroptosis and ER stress [9, 35, 40]. Targeting lipid droplet biogenesis, neutral lipid storage or lipolytic mobilisation may collapse this buffering capacity.

Silencing of LPCAT2 reduces lipid droplet accumulation and restores sensitivity to gemcitabine [41]. Inhibition of lipid droplet formation increases lipotoxic stress and sensitises PDAC cells to chemotherapy‐induced apoptosis [9, 35, 40]. Conversely, enforced lipotoxicity or disruption of neutral lipid sequestration may elevate lipid ROS beyond tolerable thresholds.

However, lipid droplets are integrated into broader metabolic networks. Suppression of storage may increase uptake or redirect carbon into alternative membrane pools, necessitating multi‐node targeting strategies.

### 8.6 Exploiting Ferroptosis Vulnerability and Autophagy Dependence

In PDAC, chemoresistant states are frequently associated with membrane MUFA enrichment, PUFA depletion and elevated GPX4 activity, which collectively reduce susceptibility to lipid peroxidation [9, 35, 45].

Multiple regulators modulate this ferroptotic threshold through control of lipid ROS generation or detoxification. Dipeptidyl‐peptidase‐4/CD26 (DPP4) promotes ferroptosis through interaction with NADPH oxidase‐1 (NOX1), forming a DPP4–NOX1 complex that drives lipid peroxidation and oxidation of polyunsaturated membrane phospholipids [25]. FSP1 acts as a lipid‐ROS detoxification system, and inhibition or genetic depletion of FSP1 restores ferroptosis sensitivity and can resensitise tumours to chemotherapy; pharmacological inhibitors such as iFSP1 and icFSP1 enhance ferroptosis by disrupting this antioxidant pathway [29].

Alterations in lipid metabolic enzymes further shift ferroptosis susceptibility. ACOT8 knockdown restores PUFA abundance, increases lipid peroxidation and enhances drug sensitivity [35, 45]. Similarly, inhibition of SCD1, which drives MUFA synthesis, sensitises PDAC cells to ferroptotic stress [9, 21, 23, 26, 35].

Additional metabolic and stress‐response pathways reinforce ferroptosis tolerance. Disruption of autophagy‐related proteins, including p62 and core ATG components, can resensitise resistant PDAC cells to therapeutic stress [5]. Loss of GSTP1 induces broader metabolic remodelling, perturbing redox homoeostasis and lipid metabolic flux, thereby altering the balance between fatty‐acid synthesis and oxidation [11, 50].

Restoring ACSL4 activity or counteracting CAF‐mediated signalling can also enhance chemotherapy response by re‐sensitising tumour cells to ferroptotic cell death [47].

Therapeutically, combination strategies pairing ferroptosis inducers with chemotherapy or lipogenic inhibitors show improved preclinical efficacy by simultaneously destabilising lipid buffering capacity and imposing oxidative stress on stress‐tolerant tumour subpopulations [35, 58]. However, ferroptosis induction alone may be insufficient where compensatory mechanisms such as increased lipid uptake or reinforcement of antioxidant defences remain active.

The reported alterations in lipid metabolism, the drugs associated with each and their contributions with respect to cytotoxic chemotherapy resistance are summarised in Table 1.

**Table 1:** Lipid metabolic mechanisms associated with resistance to different therapeutic modalities in PDAC.

Drug	Lipid mechanism	Resistance‐associated effect	References
Gemcitabine	LPCAT2‐mediated lipid droplet formation	Increased lipid storage and gemcitabine resistance	Lin et al. [41]
Gemcitabine	FSP1/GPX4‐associated ferroptosis defence	Reduced lipid peroxidation and resistance	Jin et al. [29], Gao et al. [44]
Gemcitabine	TGF‐β2‐driven lipid rewiring	Reduced gemcitabine cytotoxicity	Ma et al. [59]
Platinum‐based agents	FASN/lipogenic metabolism	FASN inhibition partially restores drug sensitivity	Liu et al. [5], Zhang et al. [28], Bader et al. [32], An et al. [34]

### 8.7 The Failure of Single‐Node Targeting

The limited clinical success of lipid‐targeted therapies reflects several factors:

- Metabolic plasticity and rapid flux redistribution.
- Systemic toxicity due to shared lipid pathways in normal tissues.
- Intratumoural heterogeneity and minor resistant subpopulations.
- Stromal and immune metabolic support.
- Absence of flux‐guided patient stratification.

Inhibition of a single enzymatic node often triggers adaptive compensation through uptake, remodelling or substrate switching [9, 76]. Lipid networks are highly interconnected. Therefore, durable therapeutic responses likely require combinatorial strategies targeting synthesis, uptake, storage and ferroptosis defence simultaneously.

### 8.8 Flux‐Guided and Phenotype‐Based Stratification

A persistent translational gap exists between lipid vulnerabilities identified in preclinical models and durable clinical benefit. This disconnect likely reflects inadequate stratification by lipid metabolic subtype and failure to account for flux plasticity.

High‐resolution lipidomic phenotyping integrated with isotope tracing may enable identification of lipogenic, lipid‐scavenging or ferroptosis‐resistant subtypes. Such stratification would permit rational pairing of chemotherapy with lipogenesis inhibitors, ferroptosis inducers or uptake blockers according to functional metabolic state rather than static abundance metrics.

Membrane‐centric and flux‐guided strategies, particularly those destabilising saturation balance, lipid storage buffering or cholesterol‐dependent signalling platforms, may provide greater leverage than upstream oncogenic inhibition alone.

## 9 Limitations, Experimental Gaps and Future Directions

Despite substantial mechanistic insight into lipid metabolic reprogramming in PDAC, significant experimental and translational gaps remain. Many conclusions are derived from in vitro systems or limited patient cohorts, and functional integration of lipidomics with flux analysis and clinical stratification remains incomplete.

### 9.1 Limited PDAC‐Specific Lipidomic Data Sets

Comprehensive, high‐resolution lipidomic data sets specific to PDAC remain scarce. Existing studies often analyse limited lipid classes or rely on bulk tumour samples without resolving intratumoural heterogeneity [5, 6, 9].

Many data sets are cross‐sectional rather than longitudinal, limiting interpretation of dynamic lipid remodelling during therapy. Longitudinal sampling before, during and after chemotherapy would better define adaptive trajectories and resistance emergence [77, 78, 79, 80].

Standardisation across platforms also remains a challenge. Variability in extraction protocols, ionisation methods, annotation pipelines and quantification strategies complicates cross‐study comparison and meta‐analysis. Harmonised methodological frameworks are required to ensure reproducibility and translational relevance.

### 9.2 Insufficient Integration of Flux‐Resolved Data

Although isotope tracing provides mechanistic resolution, in vivo flux studies in PDAC patients remain limited [52, 69, 71, 72]. Clinical tracer studies are constrained by ethical considerations, limited tracer selection, infusion duration and sampling accessibility.

Most flux analyses are performed in cell lines or xenograft models that may not accurately recapitulate stromal–tumour metabolic exchange or physiological substrate availability [52, 69, 71, 72]. Tumour lipid metabolism in vivo frequently diverges from cell culture conditions, particularly regarding acetate utilisation and uptake‐versus‐synthesis balance.

Future studies must prioritise patient‐derived models, organoids co‐cultured with stromal components and, where feasible, clinical isotope infusion studies to capture physiologically relevant metabolic routing.

### 9.3 Intratumoural Heterogeneity and Spatial Zonation

As discussed earlier, PDAC exhibits pronounced metabolic heterogeneity shaped by hypoxia, nutrient gradients and stromal composition [5, 9, 54, 55]. Bulk lipidomic analyses obscure minority resistant subpopulations that may drive relapse.

Spatial and single‐cell lipidomic approaches are technically demanding and not yet widely implemented [9, 67]. Integration of lipidomics with spatial transcriptomics, proteomics and imaging mass spectrometry is required to map metabolic zonation and identify resistance niches.

Failure to account for heterogeneity likely contributes to the translational gap between metabolic vulnerabilities identified in preclinical systems and limited clinical benefit.

### 9.4 Incomplete Consideration of Tumour–Stroma and Immune Coupling

Many therapeutic strategies target tumour‐intrinsic lipid metabolism without fully accounting for metabolic coupling with CAFs and immune cells [8, 20, 51]. Stromal lipid transfer, immune cell lipid accumulation and extracellular vesicle‐mediated lipid exchange contribute to ecosystem‐level resistance.

Experimental models that exclude stromal and immune compartments underestimate the resilience of lipid metabolic networks. Co‐culture systems, organotypic models and in vivo studies are necessary to dissect intercellular lipid exchange and identify vulnerabilities that persist in the TME.

### 9.5 Challenges in Clinical Translation

Systemic toxicity represents a major barrier to lipid‐targeted therapy, as lipid metabolic pathways are essential in normal tissues [9, 76]. Selective tumour targeting remains difficult, particularly for enzymes such as FASN, ACC or HMGCR.

Metabolic plasticity further limits efficacy. Inhibition of one pathway often triggers compensatory activation of uptake, remodelling or alternative substrate utilisation [9, 76]. Without flux‐informed stratification, patient selection remains empirical rather than mechanism‐based.

Biomarker development also remains incomplete. Although lipidomic signatures in tumour tissue and serum show promise as diagnostic, prognostic or treatment‐response markers, prospective validation in large clinical cohorts is lacking [23].

### 9.6 Future Directions

Progress in overcoming lipid‐driven chemoresistance will require:

- Integration of species‐resolved lipidomics with isotope tracing and flux modelling.
- Spatial and single‐cell resolution to capture metabolic heterogeneity.
- Longitudinal patient sampling during therapy.
- Mechanism‐guided patient stratification based on functional lipid phenotypes.
- Combination strategies targeting synthesis, uptake, storage and ferroptosis defence.
- Incorporation of stromal and immune metabolic interactions into experimental design.

Critically, lipid metabolism in PDAC should be conceptualised not as a secondary metabolic adaptation but as a structural and regulatory system encoding tumour survival under therapeutic stress. Functional interrogation of lipid flux, saturation balance and membrane architecture must replace reliance on static abundance measurements.

## 10 Conclusion

Chemoresistance in PDAC cannot be adequately explained by mutational burden or isolated pathway activation. Instead, it reflects coordinated non‐genetic metabolic rewiring in which lipid metabolism functions as a central structural and signalling axis. Within the hypovascular, desmoplastic TME, PDAC cells redistribute carbon flux across glucose, glutamine, acetate and exogenous fatty acid pathways to sustain membrane biogenesis, redox balance, lipid storage and survival signalling under chemotherapeutic stress.

Resistance to cytotoxic chemotherapy is therefore encoded not simply through total lipid abundance, but through systems‐level reorganisation of lipid species composition, fatty acid saturation balance, membrane architecture, subcellular lipid compartmentalisation and metabolic flux routing. These adaptations stabilise stress‐tolerant, stem‐like and ferroptosis‐resistant states while maintaining membrane integrity and signalling robustness. The coexistence of de novo lipogenesis, exogenous lipid uptake, cholesterol enrichment and lipid droplet buffering reflects coordinated metabolic plasticity rather than biochemical inconsistency.

Bulk lipidomics and steady‐state metabolite measurements are insufficient to resolve these adaptive states. Integration of species‐resolved lipidomics with stable isotope tracing, flux analysis and spatial approaches is required to identify functional metabolic vulnerabilities and characterise heterogeneous chemoresistant subpopulations within the tumour ecosystem.

Therapeutically, single‐node metabolic inhibition is unlikely to produce durable responses due to compensatory flux redistribution and stromal metabolic support. Membrane‐centric and flux‐guided combination strategies targeting lipid synthesis, uptake, storage and ferroptosis defence alongside conventional chemotherapy may therefore provide a more effective framework for overcoming resistance in PDAC. The mechanistic evidence reviewed here is concentrated on cytotoxic chemotherapy and should not be extrapolated to targeted therapies or immunotherapies in the absence of direct supporting evidence.

## Author Contributions

Janeesh Plakkal Ayyappan and Revathi Valliyodu conceptualised the study. Revathi Valliyodu conducted the literature review and data curation, and drafted the initial manuscript. Janeesh Plakkal Ayyappan, Revathi Valliyodu, Diya Maria Siril, Harsha Sanil, Areej Nazarudeen, Aswathy Usha Vijayamoorthy, Mahesh Chandran Valiyavilakathuveedu Rajan., Sudhina Sufina, Chandana Yesudas, Abhirami Nalinakshan, Radhika Achuthan and Muthunayagam Vibin contributed to reviewing, editing and finalising the manuscript. All authors have read and agreed to the final version of the manuscript.

## Ethics Statement

The authors have nothing to report.

## Consent

The authors have nothing to report.

## Conflicts of Interest

The authors declare no conflicts of interest.

## Data Availability

No new data were generated or analysed in this study.
